# Patients’ views of wearable devices and AI in healthcare: findings from the ComPaRe e-cohort

**DOI:** 10.1038/s41746-019-0132-y

**Published:** 2019-06-14

**Authors:** Viet-Thi Tran, Carolina Riveros, Philippe Ravaud

**Affiliations:** 1 METHODS Team, Center for Research in Epidemiology and StatisticS (CRESS) – Université Paris Descartes INSERM (UMR 1153), 1 Place du Parvis Notre Dame, 75004 Paris, France; 2 Paris Descartes University, 12 Rue de l’École de Médecine, 75006 Paris, France; 3 Center for Clinical Epidemiology, Hôtel-Dieu Hospital, Assistance Publique-Hôpitaux de Paris (AP-HP), 1 Place du Parvis Notre Dame, 75004 Paris, France; 4 Department of Epidemiology, Columbia University Mailman School of Public Health, 22W 168th St, New York, NY USA

**Keywords:** Epidemiology, Quality of life

## Abstract

Wearable biometric monitoring devices (BMDs) and artificial intelligence (AI) enable the remote measurement and analysis of patient data in real time. These technologies have generated a lot of “hype,” but their real-world effectiveness will depend on patients’ uptake. Our objective was to describe patients’ perceptions of the use of BMDs and AI in healthcare. We recruited adult patients with chronic conditions in France from the “Community of Patients for Research” (ComPaRe). Participants (1) answered quantitative and open-ended questions about the potential benefits and dangers of using of these new technologies and (2) participated in a case-vignette experiment to assess their readiness for using BMDs and AI in healthcare. Vignettes covered the use of AI to screen for skin cancer, remote monitoring of chronic conditions to predict exacerbations, smart clothes to guide physical therapy, and AI chatbots to answer emergency calls. A total of 1183 patients (51% response rate) were enrolled between May and June 2018. Overall, 20% considered that the benefits of technology (e.g., improving the reactivity in care and reducing the burden of treatment) greatly outweighed the dangers. Only 3% of participants felt that negative aspects (inadequate replacement of human intelligence, risks of hacking and misuse of private patient data) greatly outweighed potential benefits. We found that 35% of patients would refuse to integrate at least one existing or soon-to-be available intervention using BMDs and AI-based tools in their care. Accounting for patients’ perspectives will help make the most of technology without impairing the human aspects of care, generating a burden or intruding on patients’ lives.

## Introduction

The development of wearable biometric monitoring devices (BMDs) (i.e., sensors embedded in smartphones or wearable equipment to collect physiological, biological, environmental or behavioral information) allows for remote, high-frequency and high-resolution monitoring of patients’ health outside the hospital.^1–3^ Coupled with the progress of artificial intelligence (AI), the thousands of data points collected from BMDs may help in informing diagnosis, predicting patient outcomes, and helping care professionals select the best treatment for their patients.^4,5^ These two technical revolutions have generated a lot of hope and “hype,” and myriad digital and AI-based tools for healthcare have been developed.^6–8^

Today, AI can outperform medical practitioners in the analysis of skin lesions, pathology slides, electrocardiograms or medical imaging data.^9–12^ Continuous glucose monitoring systems combined with closed-loop insulin delivery systems can improve type 2 diabetes mellitus control.^13^ Multiple AI algorithms using data from BMDs are being tested to detect unknown disease, predict patient outcomes and provide reactive guidance or proactive interventions.^14–18^ Despite these good preliminary results, the real-world effectiveness of such interventions that occur outside of hospitals is still uncertain and will depend on patients’ engagement, uptake and adherence to these interventions.^19^

The literature on patients’ views of the use of BMDs and AI in healthcare is scarce and relies mainly on context-specific studies, with limited generalizability, and on evaluations of specific interventions reported in some trials evaluating these technologies, which do not reflect whether patients would have engaged in these interventions outside of the research context.^20,21^ In this study, we aimed to describe chronic patients’ perceptions of the use of BMDs and AI-based tools in healthcare and assess their readiness to integrate such technologies in their care.

## Results

### Participants’ characteristics

A total of 1183 patients with chronic conditions [861 (73%) female] participated in the study between May and June 2018 (participation rate: 48%) (Table 1). The mean age was 49.7 years (SD = 14.5). Patients’ conditions included diabetes (*n* = 121), asthma (*n* = 77), rheumatologic conditions (*n* = 367), neurological disorders (*n* = 234) and cancer (*n* = 107). A total of 649 (54%) participants had multimorbidity (mean number of conditions 2.5 [SD = 2.4]). In total, 590 (50%) participants reported using e-health or m-health tools for health [smartphone apps for 246 (21%), wellness wearable devices for 61 (5%), medically prescribed wearable device (e.g., continuous glucose monitoring tool) for 50 (4%), and health internet services (eg, online appointment tools) for 190 (16%)].

**Table 1:** Participant’s characteristics (*n* = 1183)

Characteristic	Raw data	Weighted data
Age (years)—Med (IQR)	50 [38–62]	56 [43–67]
Female sex—*n* (%)	861 (73)	641 (54)
Educational level—*n* (%)		
Lower education	62 (5·2)	115 (9·7)
Middle school or equivalent	135 (11·4)	667 (56·4)
High school or equivalent	184 (15·6)	163 (13·8)
Associate degree	266 (22·5)	104 (8·8)
Undergraduate or graduate degree	536 (45·3)	134 (11·3)
Number of chronic conditions—Med (IQR)	2 [1–3]	2 [1–3]
Multimorbidity—n (%)	649 (55)	703 (59)
Conditions—n (%)		
Asthma	77 (6)	72 (6)
Chronic obstructive pulmonary disease	23 (1)	35 (3)
Other respiratory diseases	111 (9)	118 (10)
Diabetes	121 (10)	192 (16)
Thyroid disorders	128 (11)	128 (11)
High blood pressure	137 (12)	190 (16)
Dyslipidemia	54 (5)	88 (7)
Other cardiac or vascular diseases	111 (9)	143 (12)
Chronic kidney diseases	79 (7)	101 (8)
Rheumatologic conditions	367 (31)	373 (31)
Systemic conditions	113 (10)	80 (7)
Digestive conditions	169 (14)	132 (11)
Neurological conditions	234 (20)	252 (21)
Cancer (including blood cancer)	107 (9)	108 (9)
Depression	77 (6)	76 (6)
Time since first chronic condition diagnosis (years)—Med (IQR)	14 [6–26]	16 [7–29]
Previous use of e-health or m-health tools—*n* (%)	590 (50)	604 (51)
Type of e-health/m-health tools previously used—*n* (%)		
Health smartphone apps	246 (21)	273 (24)
Wearable devices for wellness	61 (5)	58 (5)
Wearable devices prescribed by doctors	50 (4)	49 (4)
Health internet services	190 (16)	188 (16)

Weighted data were obtained after calibration on margins for sex-specific age categories and educational level with data from a national census describing the French population self-reporting at least one chronic condition

*IQR* interquartile range

### Patients’ perceptions of the use of BMDs and AI in healthcare

**Fig. 1:**
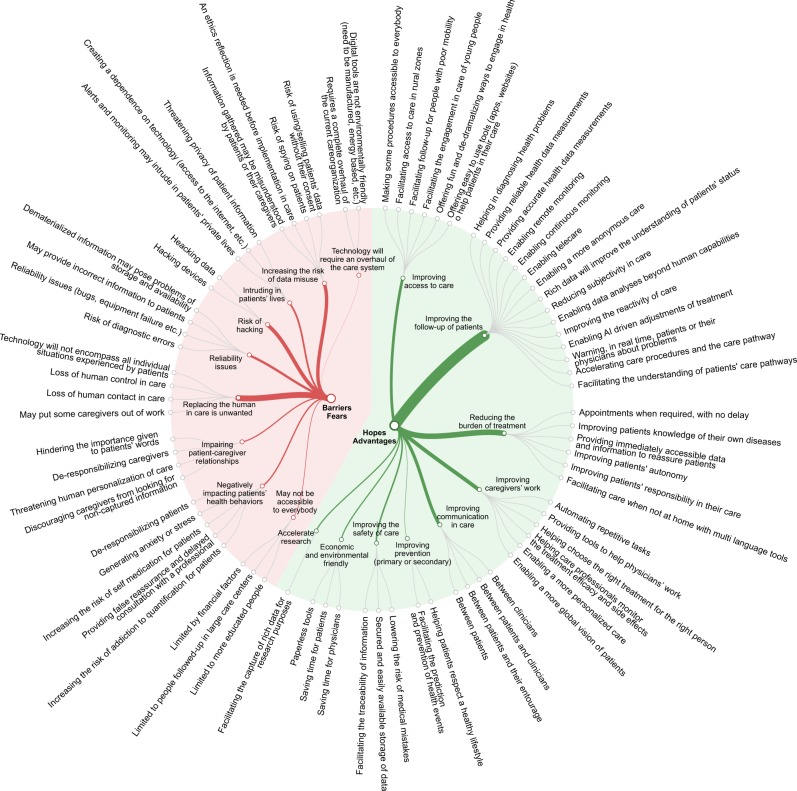
Patients’ perceived benefits and risks for the use of digital technologies and AI in healthcare. Categories presented were defined by thematic analysis of patients’ open-ended answers. The line thickness represents the number of participants who elicited each theme

After calibrating the dataset to obtain estimates representative of the French population of patients with chronic conditions, we found that 47% of participants considered BMDs and AI as a great opportunity (rating > 7/10) (IQR of opportunity ratings: [5-9]) (Supplementary Table 1). With the open-ended questions, patients identified 47 potential benefits of the use of technology in healthcare. They believed that it could improve their follow-up and the reactivity of care (55%), reduce their burden of treatment (23%) or facilitate physicians’ work (eg, by automating repetitive tasks) (21%) (Fig. 1, Table 2, Supplementary Table 2). For example, a patient described these new technologies as *“the only way [for a physician] to simultaneously take into account all multiple parameters necessary to adjust diabetes treatment: insulin sensitivity, duration of action, blood sugar levels, physical activity, continuous measurement…”* (35-year-old man reporting diabetes and Hashimoto’s thyroiditis).

**Table 2:** Potential benefits reported by patients regarding the use of biometric monitoring devices (BMDs) and artificial intelligence (AI) in their care (*n* = 985)

Categories and example of quotes	% of patients eliciting the idea (Raw)	% of patients eliciting the idea (Weighted)
Improving access to care “Care can happen everywhere. [This will help in] adjusting treatment remotely and preventing complications.” (41-year-old woman with a digestive condition)	15	12
Improving the follow-up of patients with chronic conditions “Connected applications and tools will help patients in monitoring their symptoms by guiding their observations and informing them. This will reassure them, help them to better know themselves and their diseases. This will help their caregivers in their diagnoses.” (30-year-old woman with chronic ulcerative colitis)	61	55
Reducing the burden of treatment “The development of remote could make life easier for patients and save doctors' time, especially in rural areas. This will free-up emergency services. … It could also reduce the number of "duplicate" procedures by facilitating the—regulated—access by all caregivers to the patient’s data, thus saving time for everyone.” (61-year-old man with a thyroiditis disease and polyps)	31	23
Improving caregivers’ work “Technology will help avoiding missing … the diagnosis of rare diseases for which the first symptoms are not always obvious. This may help doctors who are not specialists in these rare diseases.” (62-year-old woman with Hashimoto’s thyroiditis and interstitial pneumonia)	21	21
Improving communication in care “Precise data will complement what the patient is saying …. It will replace questionnaires and box ticking.” (27-year-old woman with asthma)	17	12
Improving prevention of diseases (primary or secondary) “Artificial intelligence makes it possible to detect cancer earlier with image recognition.” (38-year-old woman with Hashimoto’s thyroiditis)	2	3
Improving the safety of care “Diagnosis will be faster, more accurate and with less risk of errors” (61-year-old man with a thyroiditis disease and polyps)	8	7
Economic and environmental friendly solutions for care “Reducing the storage of paper medical records will be better for the planet” (54-year-old woman with depression)	6	5
Accelerating research “Analysis of very large number of data on targeted populations will allow [researchers] to refine the possible causes of pathologies and their evolution over time without necessarily requiring the implementation of costly and sometimes dangerous clinical tests for patients.” (54-year-old man with multiple sclerosis)	6	5

Categories presented were defined by thematic analysis of patients’ open-ended answers

In contrast, 11% participants considered BMDs and AI as a great danger (rating >7/10) (IQR of danger ratings: [2–6]). With the open-ended questions, patients identified 31 potential risks for the use of technology in healthcare. They feared that it could inadequately replace human intelligence in care (28%), represent serious risks for hacking (13%), or lead to misuse of private patient data by caregivers, insurance companies, etc. (14%) (Fig. 1, Table 3, Supplementary Table 3). For example, a patient stated that: “*[we will need] to be extra careful about personal data. There are risks or drawbacks if some information is disclosed to social networks, banks, insurance or work. It will be necessary for patients to be educated on that*.” (60-year-old woman reporting rheumatoid arthritis, high blood pressure, and hypercholesterolemia).

**Table 3:** Potential risks reported by patients regarding the use of BMDs and AI in their care (*n* = 964)

Category and examples of patient’s quote	% of patients eliciting the idea (Raw)	% of patients eliciting the idea (Weighted)
Accessibility issues “The internet network outside of major urban centers is lacking. Remote monitoring and data transmission require inconceivable speeds and uninterrupted power not possible in rural areas. The result will be a growing medical divide between those in cities and others” (71-year-old man with prostate cancer)	3	3
Negatively impacting patients’ health behaviors “[I fear that some patients] will feel self-sufficient and neglect their real medical follow-up” (31-year-old woman with hypothyroidism)	7	7
Impairing patient-caregiver relationships/Automation complacency “[I fear that caregivers will] rely too much on technology although it is not adapted in some situations. They will believe less [in] patients’ words and think that technology is superior evidence.” (51-year-old woman with high blood pressure)	6	6
Replacing the human in care is unwanted “Nothing beats a ‘human’ opinion to better take into account patients' feelings about their illness.” (31-year-old woman with a Hashimoto’s thyroiditis)	33	28
Reliability issues “Making people dependent on technology that require very complex infrastructures (networks, datacenters, sophisticated objects, etc.) … which are often fragile and prone to failure” (37-year-old man with chronic fatigue syndrome)	13	15
Risk of hacking “risks of hacking, risk of fraudulent use of medical data” (66-year-old man with chronic ulcerative colitis)	20	13
Intruding in patients’ lives “What is the real use of the data? Can I have a right of access to certain data that I wish to keep confidential (sexual orientation...)?” (45-year-old man with chronic heart failure)	9	7
Increasing the risk of data misuse “Unwanted access to personal data to people not subject to medical confidentiality, eg, insurance, bank, employers....” (69-year-old woman with Crohn’s disease)	19	14
Technology will require an overhaul of the care system “This implies that professionals will need to be ready and able to provide a real follow-up after [alerts from BMDs], and that they know how to react according to the information.” (30-year-old man with vitiligo)	1	1

Categories presented were defined by thematic analysis of patients’ open-ended answers

Overall, 20% participants considered that the potential benefits of technology greatly outweighed its potential dangers (opportunity > 7/danger < 3), whereas only 3% felt that negative aspects outweighed potential benefits (opportunity < 3/danger > 7) (Supplementary Table 1).

### Assessment of patients’ readiness to integrate interventions using BMDs or AI in their own care

**Fig. 2:**
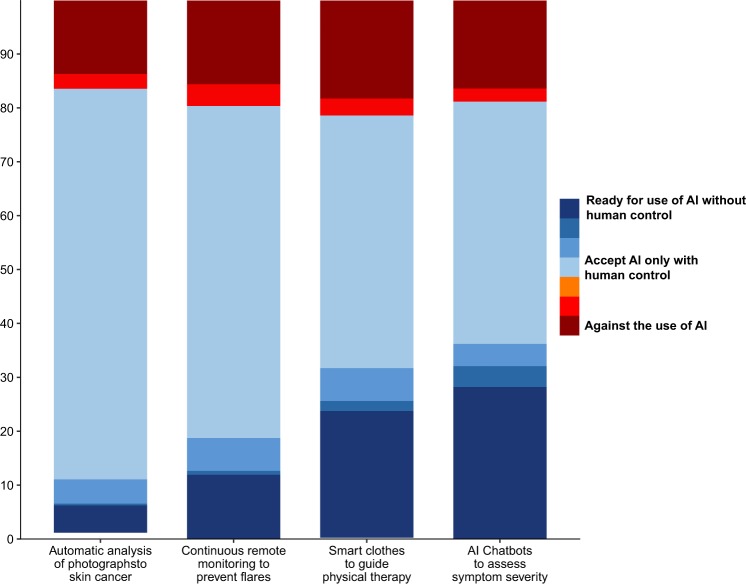
Aggregated answers to the 4 vignettes evaluating patients’ readiness to integrate specific biometric monitoring devices (BMDs) and AI-based interventions in their care (*n* = 1176). The 4 situations evaluated were the use of (1) patients’ skin photographs and AI to screen for skin cancer rather than consultations with a dermatologist;^10,22^ (2) wearable sensors for continuous and real-time monitoring and the analysis of collected data by AI to predict flares of their chronic conditions rather than usual follow-up (doctor visits, tests, etc.);^14^ (3) a smart shirt and AI to guide physical therapy rather than visits to a physiotherapist;^23^ and (4) an AI chatbot to help patients determine how urgent their problems are rather than calling an emergency telephone number.^24^ Estimates were obtained from the weighted dataset after calibration on margins for sex-specific age categories and educational level with data from a national census describing the French population self-reporting at least one chronic condition

Figure 2 and Supplementary Fig. 1 present patients’ readiness to accept four available interventions that use BMDs and AI in healthcare: (1) patients’ skin photographs and AI to screen for skin cancer;^10,22^ (2) wearable sensors for continuous and real-time monitoring and the analysis of data collected by AI to predict flares of their chronic conditions;^14^ (3) a smart shirt and AI to guide physical therapy;^23^ and (4) an AI chatbot to help patients determine how urgent their problems are.^24^ Approximately 20% of patients with chronic conditions were opposed to the use of BMDs and AI-based tools in their care in all presented situations. This proportion ranged from 17% for the use of patients’ skin photographs and AI to screen for skin cancer to 21% for the use of a smart shirt and AI to guide physical therapy. Accordingly, about 80% of participants were ready for the use of technology in their care. However, only a fraction of these patients were ready for the use of AI without human control (from 10% for the use of patients’ skin photographs and AI to screen for skin cancer to 36% for the use of AI chatbots to assist in determining how urgent their problems were).

**Fig. 3:**
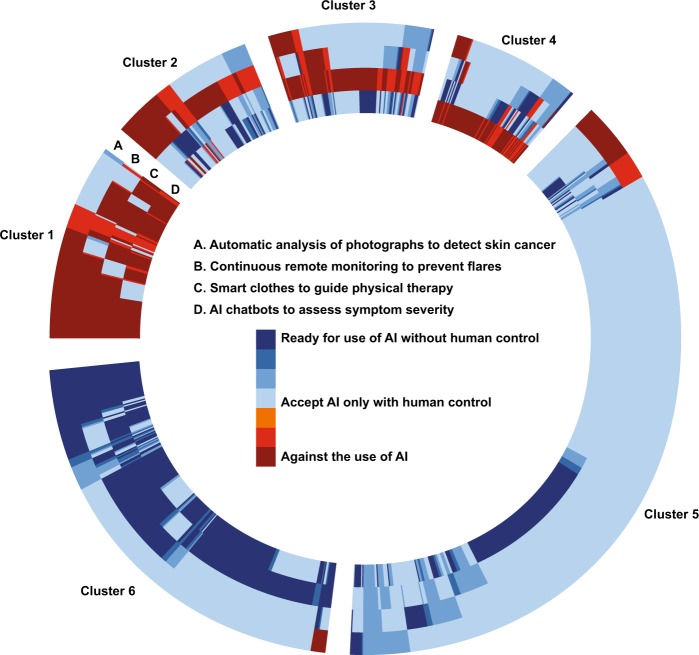
Patient profiles of readiness to integrate specific digital technologies and AI interventions in their care (*n* = 1176). Each radius of the circle represents a patient and his/her answers to the 4 vignettes. Patients were grouped by the similarity to their answers to the 4 vignettes using a k-means algorithm accounting for the weights of the calibrated dataset. Participants with missing data were dropped from analysis

We identified six clusters—or patient profiles—regarding patients’ readiness for the interventions described in the four vignettes (Fig. 3). First, 13% of participants were globally against any use of BMDs and AI (Cluster 1). Second, 22% of participants would refuse the use of BMDs and AI in one of the different situations (Clusters 2–4). Thus, only 65% of patients would agree with the integration of all interventions presented in their care. Among them, 65% (41% of the total population) would only accept BMDs and AI if their use was controlled by humans (Cluster 5), while 35% (22% of the total population) were ready for some level of automation in their care, even without human control, especially for the use of smart clothes and AI to guide physical therapy or for AI chatbots to answer emergency calls (Cluster 6). Only 3% of patients would agree with full automation of care processes for all four vignettes presented.

We found no clear association between patients’ demographic or clinical characteristics and their readiness to use BMDs and AI-based tools in their care (Supplementary Table 4).

## Discussion

In this study, we report two experiments to document the perception of patients with chronic conditions on the use of BMDs and AI in care. Only 50% of patients felt that the development of digital tools and AI in healthcare was an important opportunity and 11% considered it a danger. In particular, patients feared that the misuse of technology would lead to unwanted replacement of humans and threaten the humanistic aspect of health and care. In the second part of our study, we showed that 35% of patients would refuse to integrate at least one existing or soon-to-be available interventions using BMD- and AI-tools in their care. In addition, only a minority of participants were ready to integrate fully automatic AI-based tools in their care. Our results may explain the high drop-out of participants in the first large-scale implementations of digital monitoring strategies (90% incomplete follow-up for MyHeart Counts and 55% incomplete follow-up data for the Healthy Pregnancy Research Program).^25–27^

Our results highlight that patients intuitively think that AI should help clinicians “predict” outcomes, but that decisions, actions, and recommendations should remain a human task. Technology would be like as a “driver assistance” for clinicians.^12^ Even among patients who were the most ready for the use of technology in their care, they would only see AI as a complement—and not as replacement—for human care for situations related to sensitive topics (cancer) or which involved lasting interventions (monitoring for chronic conditions).

Our study has several strengths. As of today, it is the most comprehensive description of patients’ perceptions of the potential benefits and risks regarding the use of BMDs and AI interventions in healthcare and their readiness to integrate these technologies in their own care. Our findings were strengthened by the use of robust methods for both the quantitative and qualitative part of our study. Vignettes have been shown to accurately reflect participants’ preferences and real decisions;^28^ questionnaires with open-ended questions enabled the exploration of the broad range of patients’ perspectives regarding the use of BMDs and AI in their care without the preconceptions that could have arisen in a “classical” survey. Our study also benefited from a large sample size with wide diversity in patient characteristics (age, educational level, conditions, multimorbidity, use of m-health or e-health, etc.).

Our study has some limitations. First, despite statistical calibration, results from this study must be extrapolated with caution. Indeed, primary data were from a population of patients engaged in a citizen science initiative to accelerate research on their chronic conditions, so they may be more enthusiastic to use technology in healthcare. Second, we assessed whether patients would be ready to use digital tools and AI in their care by using clinical vignettes that were voluntarily simple. Especially, the vignettes did not detail the exact modalities of the use of technology (e.g., How many consultations could be prevented? How many alerts would the patient and/or his/her physician receive? Who would store and/or have access to collected data?). We chose this simple format because we aimed at getting the “feeling” of whether patients were ready or not for the use of BMDs and AI in their care rather than just assessing how they would accept specific interventions. Moreover, in practice, the decision to use digital tools or AI for the care of a given patient would require a discussion between the patient and the physician(s) on perceived advantages, barriers, and fears. As for any therapeutic intervention, the decision is not just that of the patient but rather a shared decision-making process with the patient–clinician dyad.

The literature on patients’ perceptions of the use of BMDs and AI in care is scarce. First, patient-reported data (qualitative or quantitative) were collected in some studies evaluating digital technologies and AI-based tools.^13,29,30^ However, these results are specific to both a given intervention and a given context and do not reflect patients’ uptake of these interventions if they had to be scaled up.^20^ Second, a handful of studies have explored patients’ perceptions of wearable devices and IA outside of the context of an ongoing digital-tool evaluation study.^19–21,31–34^ However, these studies were often limited in sample size and participant diversity or focused on a specific subject. Finally, to our knowledge, only one study broadly assessed patients’ perceptions of the use of AI in healthcare. This study from Syneos Health Communications involved 800 patients with atrial fibrillation, type 2 diabetes mellitus, and breast cancer and showed that 16–19% of participants were “excited” about use of AI in healthcare and 32 to 42% were “unexcited”.^35^ Our results provide the largest and most comprehensive view of chronic patients’ perspectives of the use of these technologies in healthcare.

Healthcare systems in high-income countries such as France strive to care for patients with chronic conditions within overburdened practices and consultations constrained to short visits.^36,37^ There is a mismatch between what care systems can and need to deliver.^38^ Therefore, many clinicians, researchers and decision makers are looking to BMDs and AI to find the “magic bullet” to transform healthcare. Although the challenges of quality and safety regarding the use of AI in care have already been noted (distributional shift, automation complacency, reward hacking, unscalable oversight, etc.),^39^ the perspective of patients has often been neglected or forgotten. Our results emphasize that patients are not ready for fully automated care. This perspective must be taken into account to avoid unjustified AI hype and to accurately assess the potential impact of implementing BMD- and AI-based interventions, at scale. For full potential of these interventions, device manufacturers, prescribing clinicians, care organizations and regulation authorities will need to account for patient-reported benefits and perceived risks, as we identified.

The number of studies evaluating new BMD- or AI-based tools is rapidly increasing and their costs are decreasing. The current literature focuses on the technological aspects of these tools but neglects patients’ perspectives of their use in healthcare. In this paper, we showed that most patients would agree to use these new technologies in their care if controlled by human caregivers. These findings call for a novel reflection about how technology should be integrated in care processes to avoid a negative impact on patient care, the generation of unnecessary burdens or the intrusion in their lives.

## Methods

### Design

This study involved two complementary parts. First, we mixed quantitative and qualitative methods to understand the potential benefits and dangers of the use of BMDs and AI in healthcare as perceived by patients with chronic conditions. Second, we used vignettes to assess chronic patients’ readiness to integrate specific interventions involving these new technologies in their care.

### Setting and participants

Participants were recruited within the “Community of Patients for Research” (ComPaRe), an ongoing citizen science project based on an e-cohort of patients with chronic conditions, in France. Participants of “ComPaRe” are adults (>18 years old) who report having at least one chronic condition (defined as a condition requiring healthcare for at least 6 months). Patients join the project to donate time to accelerate research on their conditions by answering regular patient-reported outcomes and patient-reported experience measurements online, suggesting ideas for new research, or participating in the set-up or analysis of research projects.^40^ All participants provide electronic consent before participating in the e-cohort. ComPaRe was approved by the Institutional Review Board of Hôtel-Dieu Hospital, Paris (IRB: 0008367).

To enhance the external validity of our results, our data were calibrated on margins for sex-specific weights for age and educational level derived from national census data for the French population reporting at least one chronic condition.^41,42^ The main study results are presented with the recalibrated data. Raw results are provided as supplementary tables and figures.

### Description of patients’ perceptions of the use of BMDs and AI in healthcare

A sequential explanatory mixed-methods design was used to explore patients’ perceptions of the use of BMDs and AI in healthcare.^43^ The purpose of the sequential explanatory mixed methods design was to use qualitative methods to inform the analysis of the initial quantitative results and develop a comprehensive understanding of why patients could perceive the use of BMDs and AI in care as an opportunity or danger.

First, patients rated their perceptions of the use of these new technologies by answering two structured questions “Do you think that the increasing use of digital technologies, BMDs, and AI in healthcare is an opportunity/a danger?” Ratings were collected with numeric scales ranging from 0 (no opportunity/danger) to 10 (great opportunity/danger). Answers to these questions were described by mean (SD) scores and categorized to identify participants seeing BMDs and AI as a great opportunity/danger (rating > 7/10) or small opportunity/danger (rating < 3/10).

Then, patients supplemented their numeric evaluations with open-ended comments on the benefits and risks they perceived regarding the use of these new technologies. Open-text data were evaluated by thematic analysis without the use of a specific theoretical lens. Indeed, our study was not meant to explain but rather to inventory perceptions from participants. To summarize, our analysis involved (1) the extraction by two investigators (VTT and CR), in double, of “in vivo codes”: literal terms used by participants to explain and describe their perceptions regarding the use of BMDs and AI in healthcare; (2) the comparison of these codes in order to recognize and group those that were similar, based on the context, people and processes involved; and (3) the creation, during regular meetings between the investigators, of a consensual and stable classification for codes expressing similar domains and grouping them into larger categories.

### Assessment of patients’ readiness to integrate BMDs or AI in their own care

In a second part, we presented participants with four “vignettes”—or systematically elaborated descriptions of concrete situations aimed at examining decision-making processes^44^—about existing or soon-to-be available interventions using BMDs and AI in healthcare. The four situations evaluated were the use of (1) patients’ skin photographs and AI to screen for skin cancer rather than consultations with a dermatologist;^10,22^ (2) wearable sensors for continuous and real-time monitoring and the analysis of data collected by AI to predict flares of their chronic conditions rather than usual follow-up (doctor visits, tests, etc.);^14^ (3) a smart shirt and AI to guide physical therapy rather than visits to a physiotherapist;^23^ and (4) an AI chatbot to help patients determine how urgent their problems are rather than calling an emergency telephone number.^24^ For each of these situations, participants were asked to evaluate their readiness to switch from current care to the new intervention with the question *“If there were solid scientific evidence that the [new BMD or AI intervention] would be equivalent or better than [the current standard of care] in the given situation, would you agree to use the new intervention in your own care?”* Participants’ answers ranged from −3 (would not use the intervention) to 0 (would only use the new intervention if controlled by a human caregiver), and +3 (would use the intervention and it could replace some interventions currently implemented by human caregivers).

We assessed whether homogenous groups of patients with similar readiness to use BMDs and AI could be identified from our data. For this, we considered participants’ responses to each vignette as a continuous value ranging from −3 to +3 and clustered patients according to their answers to the four vignettes with a k-means algorithm taking into account the weighted structure of the calibrated dataset. Clusters were described by both demographic and clinical characteristics (age, sex, educational level, multimorbidity, duration since the diagnosis of the first chronic condition, and previous use of e-health or m-health technology).

Analyses involved use of R v3.3 (http://www.R-project.org, the R Foundation for Statistical Computing, Vienna, Austria).

### Reporting summary

Further information on research design is available in the Nature Research Reporting Summary linked to this article.

## Supplementary information

Supplemental Information

Nature Reporting Summary

## Data Availability

All data in ComPaRe are available to academic research teams provided a scientific protocol approved by the ComPaRe scientific committee.

## Supplementary information

**Supplementary information** accompanies the paper on the *npj Digital Medicine* website (10.1038/s41746-019-0132-y).

## Code availability

The code used to process the dataset (R version 3.3 script) is available from the corresponding author on reasonable request.

## References

[1] Topol EJ, Steinhubl SR, Torkamani A (2015). Digital medical tools and sensors. Jama.

[2] Elenko E, Underwood L, Zohar D (2015). Defining digital medicine. Nat. Biotechnol..

[3] Arneric SP (2017). Biometric monitoring devices for assessing end points in clinical trials: developing an ecosystem. Nat. Rev. Drug Discov..

[4] Fagherazzi, G. & Ravaud, P. Digital diabetes: perspectives for diabetes prevention, management and research. *Diabetes Metab*. doi:10.1016/j.diabet.2018.08.012 (2018). 10.1016/j.diabet.2018.08.012 30243616

[5] Hinton G (2018). Deep learning-a technology with the potential to transform health care. Jama.

[6] Steinhubl SR, Muse ED, Topol EJ (2013). Can mobile health technologies transform health care?. Jama.

[7] Topol EJ (2010). Transforming Medicine via Digital Innovation. Sci. Transl. Med..

[8] Wicks P (2016). It’s a long shot, but it just might work! Perspectives on the future of medicine. BMC Med..

[9] Haenssle HA (2018). Man against machine: diagnostic performance of a deep learning convolutional neural network for dermoscopic melanoma recognition in comparison to 58 dermatologists. Ann. Oncol..

[10] Esteva A (2017). Dermatologist-level classification of skin cancer with deep neural networks. Nature.

[11] Lakhani P, Sundaram B (2017). Deep learning at chest radiography: automated classification of pulmonary tuberculosis by using convolutional neural networks. Radiology.

[12] Topol EJ (2019). High-performance medicine: the convergence of human and artificial intelligence. Nat. Med..

[13] Bally L (2018). Closed-loop insulin delivery for glycemic control in noncritical care. New Engl. J. Med..

[14] Burnham JP, Lu C, Yaeger LH, Bailey TC, Kollef MH (2018). Using wearable technology to predict health outcomes: a literature review. J. Am. Med. Inform. Assoc.: JAMIA.

[15] Yates T (2014). Association between change in daily ambulatory activity and cardiovascular events in people with impaired glucose tolerance (NAVIGATOR trial): a cohort analysis. Lancet (Lond., Engl.).

[16] Pyrkov TV (2018). Extracting biological age from biomedical data via deep learning: too much of a good thing?. Sci. Rep..

[17] Gresham, G. et al. Wearable activity monitors to assess performance status and predict clinical outcomes in advanced cancer patients. *npj Digital Medicine* **1**, 27 (2018). 10.1038/s41746-018-0032-6 PMC6550281 31304309

[18] Turakhia MP (2019). Rationale and design of a large-scale, app-based study to identify cardiac arrhythmias using a smartwatch: The Apple Heart Study. Am. heart J..

[19] Lennon MR (2017). Readiness for delivering digital health at scale: lessons from a longitudinal qualitative evaluation of a national digital health innovation program in the United Kingdom. J. Med. Internet Res..

[20] O’Connor S (2016). Understanding factors affecting patient and public engagement and recruitment to digital health interventions: a systematic review of qualitative studies. BMC Med. Inform. Decis. Mak..

[21] Mosconi P, Radrezza S, Lettieri E, Santoro E (2019). Use of health apps and wearable devices: survey among italian associations for patient advocacy. JMIR Mhealth Uhealth.

[22] Fujisawa Y (2018). Deep learning-based, computer-aided classifier developed with a small dataset of clinical images surpasses board-certified dermatologists in skin tumor diagnosis. Br. J. Dermatol.

[23] Bobin, M., Amroun, H., Anastassova, M., Boukallel, M. & Ammi, M. in *IEEE International Conference on Systems*, *Man, and Cybernetics (SMC*2017).

[24] Babylon. *Babylon chatbot*, https://www.babylonhealth.com/ (2017).

[25] Krummel TM (2019). The Rise of Wearable Technology in Health Care. JAMA Netw. open.

[26] Radin, J. et al. The Healthy Pregnancy Research Program: transforming pregnancy research through a ResearchKit app. *npj Digital Medicine* **1**, 45 (2018). 10.1038/s41746-018-0052-2 PMC6550256 31304325

[27] McConnell MV (2017). Feasibility of obtaining measures of lifestyle from a smartphone app: the MyHeart Counts Cardiovascular Health Study. JAMA Cardiol..

[28] Hainmueller J, Hangartner D, Yamamoto T (2015). Validating vignette and conjoint survey experiments against real-world behavior. Proc. Natl Acad. Sci. USA.

[29] Keel S (2018). Feasibility and patient acceptability of a novel artificial intelligence-based screening model for diabetic retinopathy at endocrinology outpatient services: a pilot study. Sci Rep..

[30] Kim RH, Patel MS (2018). Barriers and opportunities for using wearable devices to increase physical activity among veterans: pilot study. JMIR Form. Res..

[31] Messer LH, Johnson R, Driscoll KA, Jones J (2017). Best friend or spy: a qualitative meta-synthesis on the impact of continuous glucose monitoring on life with Type 1 diabetes. Diabet. Med.: a J. Br. Diabet. Assoc.

[32] Daus H, Kislicyn N, Heuer S, Backenstrass M (2018). Disease management apps and technical assistance systems for bipolar disorder: Investigating the patients point of view. J. Affect. Disord..

[33] Fensli, R. & E., B. in *International Joint Conference on Biomedical Engineering Systems and Technologies (BIOSTEC* 2008). (Springer).

[34] Bergmann JH, Chandaria V, McGregor A (2012). Wearable and implantable sensors: the patient’s perspective. Sens. (Basel, Switz.).

[35] Syneos Health Communications. *The Unheard Voice*, https://syneoshealthcommunications.com/perspectives/artificial-intelligence-for-authentic-engagement.

[36] Mechanic D, McAlpine DD, Rosenthal M (2001). Are patients’ office visits with physicians getting shorter?. New Engl. J. Med..

[37] Elmore N (2016). Investigating the relationship between consultation length and patient experience: a cross-sectional study in primary care. Br. J. Gen. Pract.: J. R. Coll. Gen. Pract..

[38] Kvedar JC, Fogel AL (2017). mHealth advances clinical research, bit by bit. Nat. Biotechnol..

[39] Challen R (2019). Artificial intelligence, bias and clinical safety. BMJ Qual. Saf.

[40] ComPaRe. *Community of Patients for Research*, http://www.compare.aphp.fr (2018).

[41] Institut National de la Statistique et des etudes économiques. *La macro SAS CALMAR*, 2018).

[42] Direction de la recherche, d. é., de l’évaluation et des statistiques. L’état de santé de la population en France - RAPPORT 2017. (Ministère des Solidarités et de la Santé - République Française, Paris, 2017).

[43] Creswell, J. & Clark, V. *Designing and conducting mixed methods research*. (SAGE Publications, 2011).

[44] Alexander C, Becker H (1978). The use of vignettes in survey research. Public Opin. Q..

