# Supplementary material for: Patients’ views of wearable devices and AI in healthcare: findings from the ComPaRe e-cohort
Source: NPJ Digit Med. 2019 Jun 14;2:53. doi: 10.1038/s41746-019-0132-y (PMC6572821; doi:10.1038/s41746-019-0132-y)

Supplementary table 1: Patients' evaluation of the opportunity and danger of the use of digital technologies and AI in healthcare (n=1180). Ratings ranged from 0 (no opportunity/no danger) to 10 (high opportunity/high danger). Results are expressed as raw number – raw proportion (%) – proportion after statistical weighting (%)

	**Small opportunity (rating <3) N=74**	**Moderate opportunity (rating 3–7) N=543**	**Great opportunity (rating >7) N=563**
**Small danger (rating <3) N=340**			
Raw number	19	80	241
Raw %	2%	7%	20%
Weighted %	2%	7%	20%
**Moderate danger (rating 3–7) N=677**			
Raw number	15	406	256
Raw %	1%	34%	22%
Weighted %	1%	34%	23%
**Great danger (rating >7) N=163**			
Raw number	40	57	66
Raw %	3%	5%	6%
Weighted %	3%	4%	5%

Supplementary table 2: Patients' perceived benefits of the use of digital technologies and AI in healthcare. Categories presented were defined by thematic analysis of patients' open-ended answers

**Technology may improve access to care by**
Making some care procedures accessible to everybody
Facilitating access to care fin rural zones
Facilitating the monitoring and follow-up for persons with poor mobility
Facilitating the engagement in care of young people
Offering fun and de-dramatizing health information
Offering easy to use tools (apps, websites) to help patients in their care
**Technology may facilitate caregivers' work by**
Automatizing repetitive tasks
Providing tools to help doctors' work
Helping doctors to choose the right treatment for the right person
Helping care professionals monitor the treatment efficacy and its side effects
Enabling a more personalized care
Enabling a more global vision of patients
**Technology may improve the follow-up of patients with chronic conditions by**
Helping in diagnosing health problems
Providing reliable health data measurements
Providing accurate health data measurements
Enabling remote monitoring
Enabling continuous monitoring
Enabling telecare
Enabling a more anonymous care
Enabling "rich" data capture that will help in better understanding better patients' status
Reducing subjectivity in care
Enabling data analyses beyond human capabilities
Improving the reactivity of care
Enabling automatic (AI driven) adjustments of treatment
Warning, in real time, patients or their physicians about problems
Accelerating care procedures and the care pathway
Facilitating the comprehension of patients' care pathways
**Technology may reduce the burden of care for patients by**
Facilitating the obtention of appointments for patients
Improving patients knowledge of their own diseases
Providing immediately accessible data and information to reassure patients
Improving patients' autonomy
Improving patients' responsibility in their care
Lowering the burden of treatment (e.g. limiting the number of visits)
Facilitating care in foreign countries with tools available in multiple languages
**Technology may improve communication in care by**
Facilitating information sharing between clinicians
Facilitating information sharing between patients and clinicians
Facilitating information sharing between patients and their entourage

Facilitating information sharing between patients

Technology may offer economic and environmental friendly solutions in care by
Saving time for physicians
Saving time for patients
Using less paper

Technology may improve prevention of diseases (primary or secondary)
Helping patients respecting a healthy lifestyle
Facilitating the prediction and prevention of health events

Technology may improve the safety of care by
Lowering the risk of medical mistakes
Facilitating the secured and easily available storage of data
Facilitating the traceability of information

Technology may accelerate research
By facilitating the capture of rich data for research purposes

Supplementary table 3: Patients' perceived risks of the use of digital technologies and AI in healthcare. Categories presented were defined by thematic analysis of patients' open-ended answers

**Technology may not be accessible to everybody**<br>Lack of education will limit the access to this type of care<br>This type of care will not be available everywhere<br>Costs will limit access to this type of care for everyone<br><br>**Technology will have a negative effect on patients' health behaviors**<br>Risk of addiction to quantification for patients<br>False reassurance from digital tools may delay consultation with a professional<br>Increased risk of self medication for patients<br>Digital monitoring may generate anxiety or stress for patients<br>Technology may de-responsibilize patients<br><br>**Technology will have a negative effect on patient-caregiver relationships**<br>Digital technologies may discourage caregivers/researchers to look for other non captured information<br>Digital tools may threaten personalization of care<br>Artificial intelligence may de-responsibilize caregivers (Who will hold responsibility in case there is an error?)<br>Digital data may hinder the importance given to patients' words<br><br>**Technology may have reliability issues**<br>Risk of diagnostic errors<br>Reliability issues (bugs, equipment failure…)<br>May provide incorrect information to patients<br>Dematerialized information may pose problems of storage and availability<br><br>**Technology may intrude in patients' lives**<br>Alerts and monitoring may intrude in patients' private lives<br>Technology may hinder patients' freedom (need to have access to internet, etc.)<br>May threaten privacy of patients' information<br><br>**Technology increases the risk of data misuses**<br>Information gathered using digital tools may be misunderstood by patients or their caregivers<br>Necessitates an ethics reflexion before being used in care<br>Risk of spying on patients<br>Data collected may be used without patients' consent or sold<br><br>**Replacing human in care is unwanted**<br>May put some caregivers out of work<br>Loss of human contact in care<br>Loss of human control in care<br>Technology will not encompass all individual situations lived by patients<br><br>**Technology is vulnerable to hacking**<br>Wearable devices are at risk of hacking<br>Security risk for data gathered on patients (hacking…)

Technology will require a complete (complex and expensive) overhaul of the care system
Necessitates a complete overhaul of the current care organization
Digital tools are not environmentally friendly (need to be manufactured, energy based, etc.)

Supplementary table 4: Patients' characteristics in each cluster automatically identified from answers to the readiness vignettes (n=1176) Patients were grouped by the similarity to their answers to the four vignettes using a k-means algorithm accounting for the weights of the calibrated dataset. Participants with missing data were dropped from analysis. Patients with missing data in at least one of their vignettes' evaluations were dropped from analysis.

Characteristic	Cluster 1 N=154 "Against any use of BMDs and AI care"	Cluster 2 N=75 "Against BMDs and AI for continuous remote monitoring"	Cluster 3 N=109 "Against automation of physical therapy"	Cluster 4 N=78 "Against automatic chatbots to answer calls"	Cluster 5 N=479 "BMDs and AI only with human control"	Cluster 6 N=269 "Accept the automation of some care processes"
**Readiness score (min: 0; max: 6) – mean (SD)**						
• AI to screen for skin cancer	1.1 (0.3)	2.2 (0.3)	2.6 (0.2)	2.9 (0.2)	2.9 (0.0)	3.5(0.2)
• Continuous remote monitoring to prevent flares	0.6 (0.2)	0.5 (0.1)	2.7 (0.2)	3.0 (0.3)	3.2 (0.1)	4.3 (0.2)
• Smart clothes to guide physical therapy	0.4 (0.2)	3.8 (0.2)	0.3 (0.1)	3.8 (0.3)	3.1 (0.0)	5.8 (0.1)
• Chatbots to assess symptom severity	0.5 (0.2)	4.0 (0.3)	3.7 (0.2)	0.2 (0.1)	3.9 (0.1)	5.0 (0.2)
**Age (years) – mean (SD)**	56.4 (3.1)	52.0 (5.0)	56.5 (2.6)	53.9 (3.0)	53.7 (1.4)	54.7 (1.6)
**Female sex (%)**	37	40	60	70	50	68
**Higher education (%)**	10	20	12	14	11	10
**Number of chronic conditions – mean (SD)**	2.4 (0.3)	2.1 (0.2)	3.0 (0.4)	3.7 (0.9)	2.7 (0.2)	2.6 (0.2)
**Time since diagnosis (years) – Mean (SD)**	19.5 (2.9)	15.1 (2.1)	21.2 (3.1)	19.0 (1.8)	18.5 (1.3)	20.9 (1.6)
**Already use e-health or m-health tools (%)**	5	69	50	57	47	37

Supplementary figure 1: Aggregated answers to the 4 vignettes evaluating patients' readiness to integrate specific BMD and AI-based interventions in their care – raw data (n=1176) Estimates were obtained using raw data (without calibration).

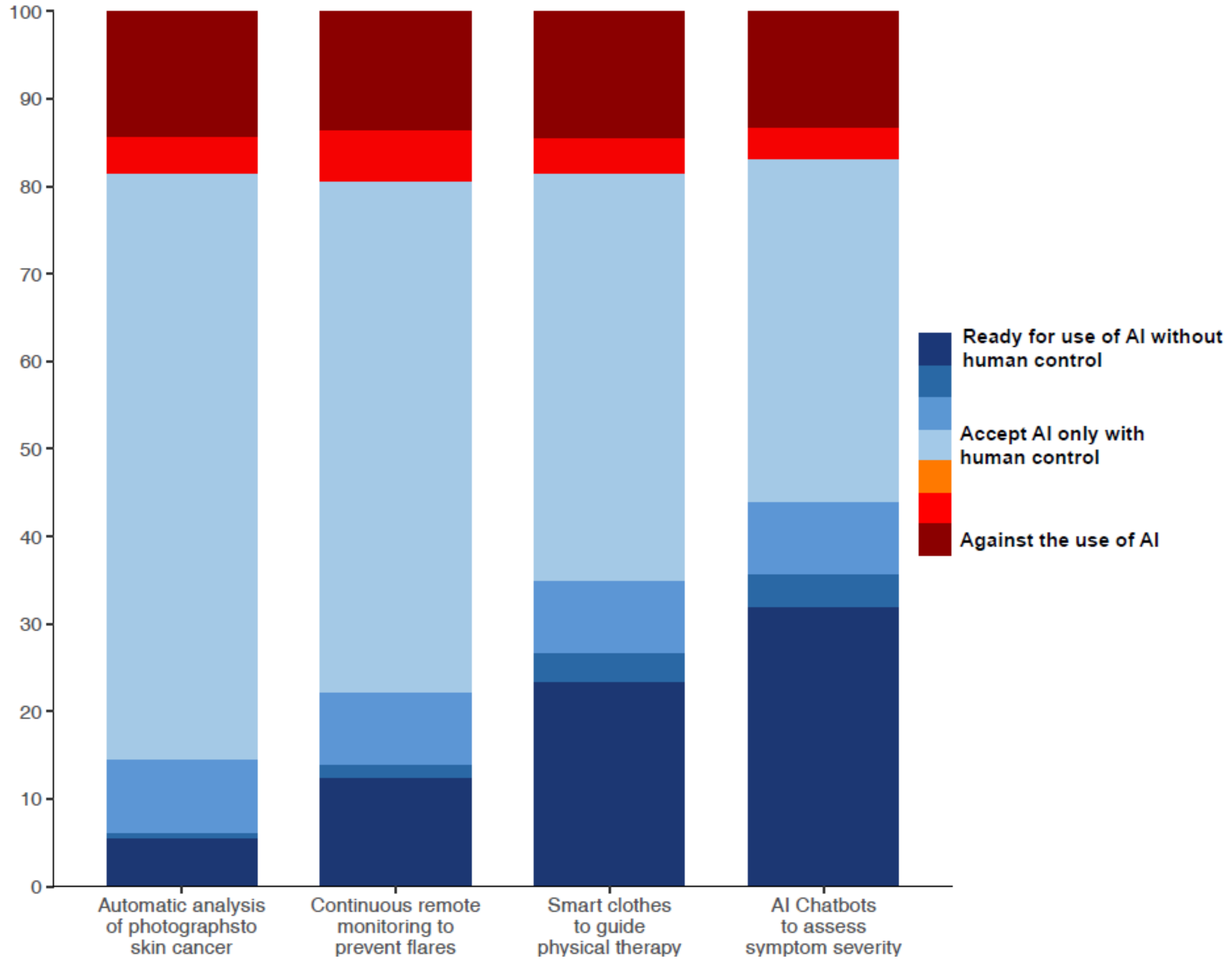

Supplement: Supplementary file 1 — Supplemental Information [file 41746_2019_132_MOESM1_ESM.pdf]
